# Rewriting paracelsus: linking chemical assessment to public health

**DOI:** 10.3389/ftox.2026.1872816

**Published:** 2026-09-02

**Authors:** Ana Fernandez-Agudo, María del Carmen González-Caballero, Jose V. Tarazona

**Affiliations:** 1 Spanish National Environmental Health Center, Instituto de Salud Carlos III, Madrid, Spain; 2 Biomedical Sciences and Public Health, Universidad Nacional de Educación a Distancia (UNED), Madrid, Spain

**Keywords:** health impact pathways (HIPs), new approach methodologies (NAMs), next-generation risk assessment (NGRA), personalized risk assessment (PRA), probabilistic risk assessment

## Abstract

Human health is continuously challenged by exposure to complex mixtures of chemicals present in food, products, and the environment. Traditional risk assessment (TRA), rooted in mid-20th-century paradigms, relies on deterministic thresholds and animal testing to define safe exposure levels. This approach, while useful for regulatory simplicity, fails to represent real-world variability, mixture effects, and lifelong events, limiting its relevance for public health protection. Here we show that a probabilistic Next-Generation Risk Assessment (NGRA) framework integrating quantitative modelling, mechanistic biology, and New Approach Methodologies (NAMs), can transform toxicological evidence into probability distributions, estimating the likelihood of adverse outcomes under realistic exposure scenarios. Central to this approach are Health Impact Pathways (HIPs), which connect molecular perturbations to population-level health indicators, bridging toxicology and epidemiology. The framework explicitly distinguishes susceptible groups, such as individuals in physiologically sensitive stages like pregnancy, from vulnerable groups, including those with pre-existing diseases or higher exposure burdens, poorly covered in TRA. By covering these groups, probabilistic NGRA enables more inclusive, relevant, and informative evaluations that align with FAIR (Findable, Accessible, Interoperable, Reusable) data principles and minimize animal testing, offering a scientifically grounded framework for improving chemical safety evaluations and public health protection.

## 1 Introduction

Humans are exposed to a variety of hazardous chemicals present in food, consumer products, occupational settings, the environment, etc. Generally, the identification and management of their potential health impact is based on adversity thresholds, following a risk assessment paradigm developed in the 1950s. This paradigm focuses on the identification of the dose that does not produce adverse effects, named toxicological threshold (Michaleas et al., 2021). This approach could be considered as the regulatory implementation of 16th century Paracelsus’ principle “*Sola dosis facit venenum*”: if “*only the dose makes the poison*”, the main focuses on the identification of the dose that does not produce adverse effects. The toxicological or adversity threshold defines, in theory, the limit between the exposure level that produce adverse effects and that without adverse consequences. In practice, this has been implemented as a level that is assumed to be safe based on the existing information, and can be refined/modified if additional information is available. The naming is different depending on the context, sector and jurisdiction, but is covered under the general umbrella of Health-Based Guidance Values (HBGV) (More et al., 2021).

This concept has been further extended developing Threshold of Toxicological Concern (TTC), extensively applied in the safety assessment in some sectors. The TTC approach uses animal toxicity data to define exposure levels below which there is a low probability of adverse health effects when specific data is limited but the chemical structure is known. Validated animal studies have led to specific TTC values for different chemical classes, guiding whether a substance requires a detailed evaluation or can be assessed using TTC. The approach has exclusion criteria, and legal requirements to generate actual toxicity data may limit its applicability (Kroes et al., 2005; More et al., 2019).

The focus on setting a level of “assumed no-effect” for each substance facilitates decisions on marketing authorizations. However, is of limited value regarding the identification of the effects associated with actual exposure levels, and the overall impact of exposure to chemicals on Public Health indicators (Chen et al., 2024).

**FIGURE 1:**
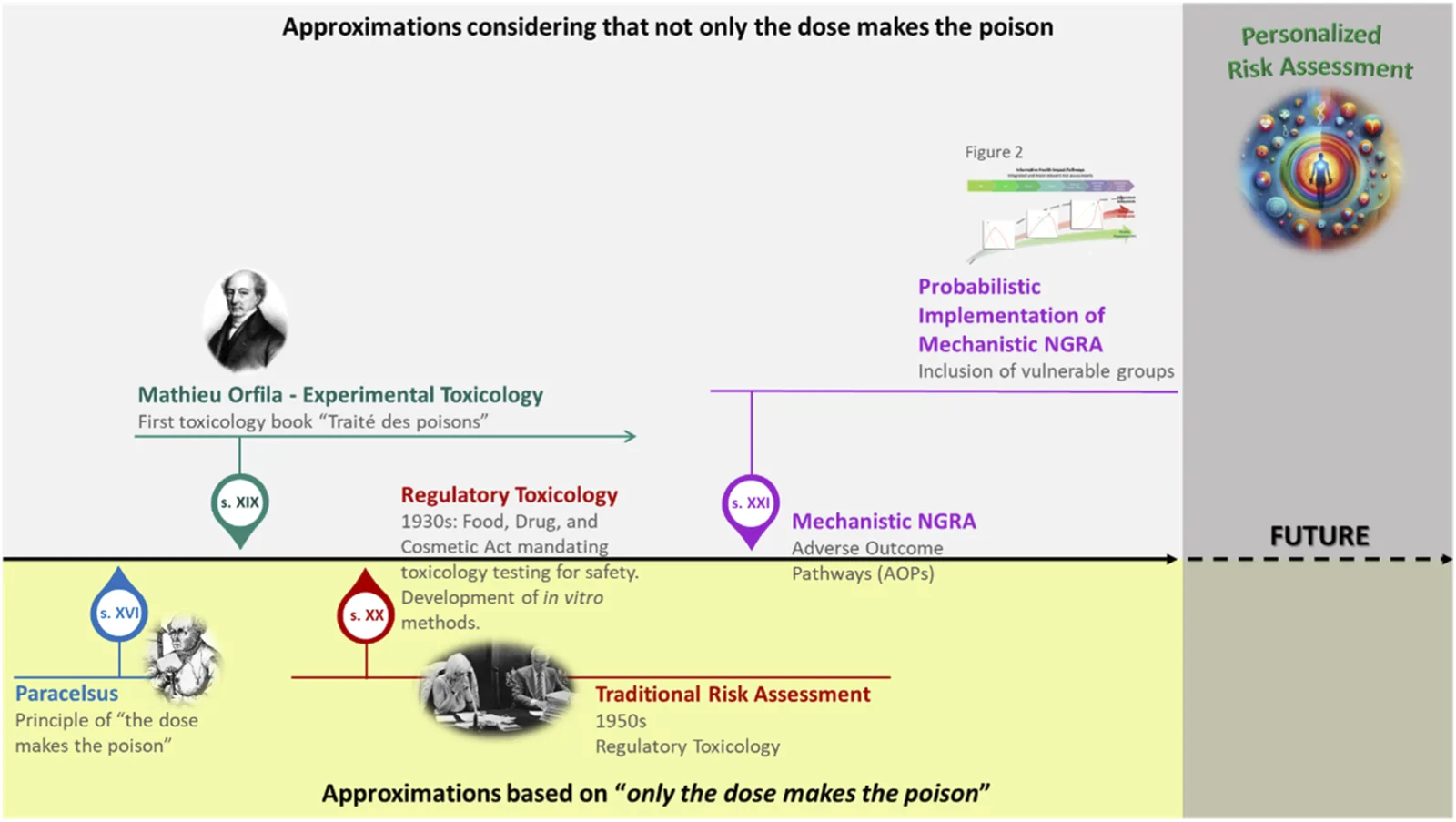
Evolution of toxicology and risk assessment toward personalization and probabilistic integration. This graphical abstract depicts the historical and conceptual trajectory of toxicology and risk assessment, from Paracelsus’ 16th-century principle *“the dose makes the poison”* to a 21st-century paradigm recognizing that *not only the dose makes the poison.* The lower panel traces the origins of deterministic toxicology, implementing Paracelsus’ principle in *Regulatory Toxicology* and *Traditional Risk Assessment (TRA)* in the mid-20th century, which defined safety through fixed thresholds derived largely from animal data. The upper trajectory represents the beginning of experimental toxicology with Orfila´s confirming the role of individual and environmental conditions in the toxicological response and the progressive transition toward human-relevant, mechanistic, and probabilistic frameworks. Beginning with *Mechanistic NGRA* and advancing through *Probabilistic NGRA*, the field now incorporates *Adverse Outcome Pathways (AOPs), Health Impact Pathways (HIPs),* and *New Approach Methodologies (NAMs)* to integrate biological mechanisms, exposure variability, and population heterogeneity. This conceptual evolution culminates in the vision of *Personalized Risk Assessment (PRA)*, a framework capable of quantifying susceptibility in physiologically sensitive groups and vulnerability in individuals with pre-existing conditions or elevated exposures. Together, these advances redefine chemical safety as a dynamic, probabilistic risk framework that bridges toxicology, data science, and public health, ensuring protection across the full spectrum of human variability.

Science has undergone a transformative revolution, with significant advancements in computing, molecular biology, and a proliferation of tools for evaluating the impact of chemical exposure. These developments have significantly enhanced our understanding of how substances interact with biological systems, particularly at the molecular and subcellular levels (Plowright et al., 2017; Zhang et al., 2025). The advent of technologies such as high-throughput screening, computational modeling, and systems biology is illuminating mechanisms of action that govern chemical-related effects on humans. A paradigm shift is incorporating 21st-century science into risk assessments, enabling more precise predictions of toxicity and fostering the integration of mechanistic insights into regulatory frameworks (National Academies of Sciences, 2017). While the new paradigm is mechanistic, the regulatory implementation is still focused on trying to estimate a Point of Departure (PoD) for identifying toxicity thresholds; e.g., using Adverse Outcome Pathways to predict if the effects observed at molecular and cellular levels are expected to progress and result in adverse outcomes, similar to observed in traditional toxicity studies in animal models (Bercu et al., 2016). The historical and conceptual evolution from Paracelsian toxicology through Traditional Risk Assessment (TRA) and Next Generation Risk Assessment (NGRA) toward probabilistic and personalized approaches is summarized in Figure 1.

In parallel, system biology and its application in system toxicology, is highlighting the need to address the complexity of chemical-health interactions. The consequences of chemical exposure are not only linked to the dose-response relationship; they are linked to the specific characteristics of each individual, the co-exposure to chemicals with similar or related modes of action, and other external factors (Vivo, 2025). We present a new framework for maximizing the capacities of the new mechanistic paradigm linking toxicological understanding with the expected consequences on human health at individual and population levels.

### 1.1 Toward a probabilistic NGRA

This manuscript proposes a novel probabilistic NGRA approach that integrates key advancements in toxicology and risk science to address the limitations of traditional risk assessment (TRA) frameworks and maximize the capacity of NGRA for informing on public health consequences. Central to this approach is the transition from threshold-based methodologies to non-threshold, pathway-based, evaluations that leverage Adverse Outcome Pathway (AOP) networks (Vinken, 2013) and advanced mechanistic insights. In this context, non-threshold pathway-based evaluations focus on characterizing the probability and magnitude of biological perturbations across exposure ranges rather than identifying a single exposure threshold assumed to separate safe from unsafe conditions. The assessment therefore considers how exposure alters key biological pathways and how these perturbations translate into changes in the probability of adverse health outcomes. By incorporating probabilistic models (Maertens, 2022), the proposed probabilistic-NGRA framework translates chemical risks into the expected consequences on health indicators under real-world exposure scenarios. While probabilistic risk assessment approaches are equally applicable to human and ecological assessments, the present manuscript focuses specifically on human health NGRA and its potential evolution toward individualized and population-based public health applications. Integrated Approaches to Testing and Assessment (IATA) provide important operational frameworks within this transition and are considered complementary tools for implementation of probabilistic NGRA.

NGRA are defined as exposure-led and hypothesis-driven approaches. The implementation of NGRA requires the integration of exposure and hazard assessments to identify the key risk drivers. Probabilistic approaches can be easily incorporated into the exposure assessment, for accounting real variability and uncertainty, e.g., in levels in food commodities and in the population diets for dietary exposure assessments (Stefano et al., 2025). Probabilistic approaches have been less explored for hazard identification and characterization. Our framework is built under the assumption that defining a toxicity threshold is not the best scientific approach for supporting informed regulatory decisions. This does not imply that thresholds lack regulatory utility. Rather, thresholds summarize complex biological processes into a single decision metric. While useful for decision-making, they do not explicitly characterize variability, uncertainty, susceptibility, vulnerability, or the probability distribution of responses across populations.

TRA focuses on setting HBGV for each chemical and route, irrespective of other exposures, stress or vulnerability factors, and then check if exposure levels are above or below the value. This approach provides limited evidence for assessing cumulative and synergistic effects. NGRA advocates for exposure led hypothesis driven approaches maximizing the use of mechanistic knowledge. New Approach Methodologies (NAMs) are pivotal to this transition. NAMs encompass a wide array of innovative technologies and methodologies that offer critical insights into the mechanisms of chemical and biological interactions without relying on animal testing. These include advanced computational models, artificial intelligence, high-throughput screening, bioactivity-based assays, toxicogenomics, and organ-on-chip technologies (Brescia et al., 2023). NAMs facilitate human-relevant toxicological evaluations while reducing reliance on animal testing (Reale et al., 2024).

Central to NGRA is the application of AOP networks, which map the biological mechanisms underlying toxicity from molecular interactions to organism effects. An AOP is an analytical construct that describes a sequential chain of causally linked events leading to an adverse health effect. AOPs are the central element of a toxicological knowledge framework supporting chemical risk assessment based on mechanistic reasoning (Tan et al., 2018). The linked events form a cascade of chemical-biological interactions with implications at different levels of biological organization (Paini et al., 2022). The AOP describes this causal chain from a molecular initiating event (MIE) triggering key events (KE) leading to an adverse outcome (AO) (OECD, 2017).

Individual AOPs rarely operate independently; interactions between pathways influence the trajectory or intensity of chemical effects. AOP networks provide a more comprehensive framework for understanding biological responses, encompassing variations in life stage, sex, target organs, and taxonomic space (Knapen et al., 2018). These networks can enhance prediction accuracy, enabling more nuanced risk evaluations across diverse scenarios.

NGRA is already replacing the traditional approach for assessing local effects such as eye/skin irritation or skin sensitization. Although proof-of-concept studies and regulatory case studies have successfully demonstrated NGRA applications for systemic toxicity, broader routine regulatory implementation remains limited across sectors and jurisdictions (Berggren and White, 2017; Baltazar and Cable, 2020; OECD, 2021a; SCCS, 2016). However, several initiatives worldwide are pacing the way for this transition. Using the EU as example, this includes research projects (e.g., the ASPIS cluster or NAMWISE), regulatory partnerships (e.g., PARC and EPAA), and policy initiatives (e.g., the ban for using animal testing for cosmetics and the ongoing European Commission Roadmap for phasing-out animal testing for chemical safety). Similar initiatives are ongoing in other jurisdictions, and international networks of regulatory bodies such as APCRA and ILMERAC offer networking possibilities for sharing experiences and harmonization.

### 1.2 Toward a new health-based paradigm

Both processes are linked to a variety of individual factors, and therefore each person is exposed to a specific combination of chemical substances (the personal exposome) (Vermeulen et al., 2020) and different individuals may have different responses to the same exposure levels to a certain chemical. Information on this variability is usually collected, but traditionally, has been condensed, and deterministic approaches (e.g., using a selected percentile (Maertens, 2022) instead of the full distribution) have been implemented to simplify the risk assessment procedures. This is no longer needed, as technological developments have simplified the management of large data sets (Oussous, 2018). Adapting risk assessment (RA) to probabilistic, pathway-based models represents a substantial methodological shift in toxicology.

## 2 Limitations of deterministic risk assessment and opportunities for probabilistic NGRA

The TRA principle underpins a threshold-based approach in which a safe level of exposure, such as an Acceptable Daily Intake (ADI) or Reference Dose (RfD), is derived by applying conservative uncertainty factors to a PoD, typically identified from animal studies (Barnes et al., 1988). This deterministic approach assumes that below this threshold, all individuals are equally protected, without discrimination of age, sex, genetic background, health status, or co-exposures to other chemicals. While this strategy provided a workable regulatory tool, it fails to capture the complexity of real-world human exposures. This interferes with the reality that adverse health outcomes emerge along a probabilistic spectrum, influenced by multiple variables including interindividual variability, life stage, co-exposures, and time-dependent processes (Maertens, 2022). The PoD is based on observed direct toxic effects in healthy animals. Mechanisms such as adaptation of functional/structural compensation remains unidentified; consequently, vulnerable groups may experience adverse effects even when general population exposures remain below regulatory thresholds, highlighting the inadequacy of this model, where a single value is expected to fit all individuals (Jager et al., 2001).

Furthermore, TRA is poorly suited for addressing low-dose effects, cumulative risks, and interactions among multiple stressors, all of which have become increasingly relevant through advances in systems biology and the exposome. Treating safety as a fixed number generates uncertainties and ignores the probabilistic nature of risk (Uusitalo et al., 2015). A great example of failure of such traditional assessments are PFAS (Attema et al., 2022), where biologically significant effects are observed at doses far below traditional thresholds.

Another challenge is the fragmented and inconsistent global regulatory scene. Safety standards vary depending on sector and jurisdiction, as seen with pesticides, biocides, or food contaminants (Devos et al., 2022), creating inefficiencies and contradictions. Initiatives such as the EU “One Substance, One Assessment” (OSOA) (EC OSOA, 2024) are steps toward harmonization, yet they remain fixed in the threshold-based, deterministic principle.

In addition, the deterministic “observational” paradigm limits the integration of mechanistic high-throughput screening, computational toxicology, human biomonitoring, and population health studies, which reveal that chemical risks are not uniform, but probabilistic and dependent to the context. Human biomonitoring, for example, demonstrates that individuals carry unique exposomes (Vermeulen et al., 2020), defined by temporally explicit chemical exposure events across their lifespans. Incorporating such variability requires probabilistic frameworks and mechanistic understanding linking each individual with the relevant risk distributions.

The proposed paradigm shift toward mechanistic NGRA is linked to probabilistic models (Maertens, 2022; Moe et al., 2022) capable of reflecting distributions of exposure and the likelihood of different responses at the individual and population level. NGRA provides the scientific mechanistic foundation, but only through probabilistic implementation can it deliver the precision and realism required for 21st-century chemical risk assessment.

## 3 Redefining risk assessment

Deterministic NGRA refers to approaches that replace animal-derived evidence with NAM-derived evidence while retaining threshold-based decision criteria and single point estimates. In this scenario, mechanistic information improves biological relevance but does not fundamentally alter the structure of risk characterization. Probabilistic NGRA differs by treating both exposure and biological response as distributions rather than fixed values. Variability, uncertainty, susceptibility, and vulnerability become explicit model components rather than implicit assumptions embedded within uncertainty factors. Accordingly, probabilistic NGRA represents not only a methodological advancement but also a conceptual shift in how chemical risks are characterized and communicated (EFSA Scientific Committee, 2024).

The limitations of the deterministic TRA model demonstrate that NGRA cannot simply replace animal testing with NAMs while maintaining the traditional reliance on static PoDs and generic uncertainty factors. Such a deterministic NGRA would still suffer from the same conceptual shortcomings of TRA.

Instead, NGRA must be implemented probabilistically. Probabilistic NGRA models uncertainty and variability, enabling chemical risk assessment to move beyond safe/unsafe assessments toward quantitative probability distributions of risk for different population groups. Through probabilistic modeling techniques, including Monte Carlo simulations, Bayesian networks, and regression-based prediction models, NGRA allows for the integration of multiple data streams to generate population-level risk estimates (Refsgaard et al., 2007). A challenge and an opportunity are the incorporation of mechanistic information to address disease-related vulnerabilities (Fernández-Martín and Tarazona, 2025); generating probability distributions across exposure ranges, demographics, and biological states. This transition reframes adversity towards a more realistic and relevant view of chemical impacts in public health.

Unlike traditional one substance assessments, NGRA supports aggregate and combined assessments of chemicals, addressing the reality of mixed exposures (Cronin et al., 2023), considering cumulative exposure scenarios and the potential for synergistic or antagonistic interactions between substances (Brasseur et al., 2023). Moving beyond dose-dependency, this new approach considers genetic and environmental factors, allowing for a complex, dynamic model of toxicity that reflects real-world scenarios and multifactorial risks (Bhuller et al., 2024). This addresses a critical limitation of previous models, which failed to capture the risks associated with combined exposures to chemical mixtures (Lagunas-Rangel et al., 2022).

A probabilistic architecture (Zou and Wen, 2023) enables NGRA to simulate both individual-level and population-level outcomes, replacing the average person assumption with dynamic models that capture variability. Importantly, probabilistic models support continuous updating and refinement as new data become available, enabling a more adaptive and predictive system. This evolution from TRA to NGRA, probabilistic NGRA, and ultimately PRA is summarized in Table 1, highlighting the progressive incorporation of mechanistic evidence, probabilistic modelling, and population heterogeneity.

**TABLE 1:** Evolution of risk assessment toward personalization and probabilistic integration.

Category	Definition	Deterministic or probabilistic	Role of NAMs	Focus	Key innovation	Relation to other categories
Traditional risk assessment (TRA)	Conventional risk assessment approach based on animal data, safety factors, and thresholds	Hazard is deterministic. Exposure can be deterministic or probabilistic	Limited use; mainly for supporting data	General “healthy” population safety	Agreements on problem formulation, exposure scenarios and acceptability thresholds for regulatory assessments	Baseline method; predecessor of NGRA
Next-generation risk assessment (NGRA)	Risk assessment using NAMs and human-relevant, mechanistic data	As above. Uses NAM-derived mechanistic information but retains threshold-based risk characterization and point estimates	Central for mechanism-based analysis	Human relevance, efficiency, ethical considerations	Uses AOPs and exposure-led hypothesis driven approaches including IATA (OECD, 2024)	Evolves from TRA; foundation for probabilistic NGRA
Probabilistic NGRA	Integration of probabilistic reasoning into NGRA using mechanistic data and HIPs	Probabilistic by design for hazard and exposure	Enables identification of vulnerable subgroups	Integration of quantitative KEs, associative mechanistic signals and HIP-based population-health indicators	Integration of susceptibility and vulnerability into probabilistic risk distributions; quantifies likelihood of adverse outcomes across subgroups	Builds on NGRA; prerequisite to Personalized RA
Personalized risk assessment (PRA)	Future approach tailoring risk estimation to individual-level variability (genetics, co-exposures, etc.)	Fully probabilistic with personalized exposure and vulnerability	Supports personalized level predictions	Individual susceptibility and real-world exposures	Personalized probability of adverse outcomes	Extends probabilistic NGRA to individual level

This table illustrates the conceptual transition from the mid-20th-century paradigm of Traditional Risk Assessment (TRA) to a future vision of *Personalized Risk Assessment (PRA)*. It introduces *Probabilistic Next-Generation Risk Assessment (NGRA)* as the pivotal intermediate framework that redefines how toxicological and exposure evidence are integrated. The progression reflects a scientific shift from deterministic, threshold-based models to probabilistic, mechanistic, and human-relevant frameworks. Probabilistic NGRA uniquely combines mechanistic data from New Approach Method*ologies (NAMs)* with probabilistic modelling to quantify variability, uncertainty, and population heterogeneity. By incorporating *Health Impact Pathways (HIPs)*, it links molecular and cellular perturbations to population-level health indicators, explicitly identifying susceptible (physiologically sensitive) and vulnerable (non-healthy or highly exposed) groups that remain unaddressed by traditional assessments. This integration paves the way for personalized, data-rich, and ethically grounded risk evaluation. Together, the categories outlined in the table chart a clear trajectory from TRA to NGRA, probabilistic NGRA, and ultimately PRA, demonstrating how chemical safety science is evolving toward adaptive, probabilistic, and individualized frameworks capable of informing real-world public health protection.

### 3.1 Integrating hazard & exposure

In the probabilistic NGRA framework described herein, both hazard characterization and exposure assessment are formulated probabilistically (Van Der Voet and Slob, 2007). The innovation of our approach is to use mechanistic hazard information, derived from NAMs, in probabilistic hazard frameworks to consider individual variability in our assessment. Mechanistic information also allows to identify under which conditions the MIE and early KEs are more likely to progress towards direct adverse health effects; including the individual characteristics that reduce recovery/compensation mechanisms or increase the probability of indirect related effects. In other words, linking mechanistic understanding with physiological knowledge, to understand why some individuals are affected and others not, and transfer this knowledge into sets of probabilistic exposure vs. likelihood for effect distributions.

### 3.2 NAMs for susceptible and vulnerable groups

TRA does account for sensitive populations through uncertainty factors and conservative assumptions. However, these approaches generally do not explicitly identify affected subgroups, quantify subgroup-specific risks, or characterize the magnitude of variability. A major strength of probabilistic NGRA is its capacity to model risk in susceptible and vulnerable populations, making interindividual variability a central component of risk characterization rather than an implicit assumption. Health risks are shaped not only by intrinsic biological variability, but also by disease status, life stage, and situational exposures.

In our approach, susceptibility refers to physiological conditions occurring within otherwise healthy individuals, such as pregnancy, infancy, puberty, or ageing, that may modify the response to chemical exposure. In contrast, vulnerability refers to conditions associated with pre-existing disease, impaired physiological function, socioeconomic factors, or unusually high exposure burdens (Fernández-Martín and Tarazona, 2025). Both susceptibility and vulnerability influence the probability that a given exposure will result in adverse health effects.

Probabilistic NGRA allows these factors to be incorporated directly into risk models. Susceptibility may be represented through age-specific physiology, developmental stage, hormonal status, altered toxicokinetics, or genetic determinants. Vulnerability may additionally include disease-associated toxicodynamic alterations, impaired compensatory mechanisms, medication use, chronic inflammation, socioeconomic determinants of exposure, or combinations thereof. These determinants can be parameterized using subgroup-specific probability distributions within toxicokinetic, toxicodynamic, exposure, and health outcome models (EFSA Scientific Committee, 2024). Probabilistic simulation techniques, including Monte Carlo and Bayesian approaches, can integrate these distributions and continuously refine risk estimates as new evidence becomes available (Trang et al., 2017).

The integration of NAM-derived mechanistic information further strengthens this framework by supporting identification of the biological determinants underlying susceptibility and vulnerability. For example, toxicokinetic vulnerability may arise from reduced metabolic clearance, altered transporter activity, impaired renal function, or physiological changes associated with pregnancy. In contrast, toxicodynamic vulnerability may originate from altered receptor expression, chronic inflammatory states, pre-existing disease processes, or impaired compensatory responses. NAM platforms including organoids, toxicogenomic assays, receptor-based screening systems, and disease-specific cellular models can contribute to the characterization of these vulnerability profiles. A practical implementation may combine probabilistic exposure distributions with physiologically based toxicokinetic (PBTK) models, NAM-derived concentration-response functions, and quantitative key-event relationships to generate probability distributions for specific health outcomes.

Developmental neurotoxicity illustrates the value of this approach. NAM data generated from neural progenitor cells, brain organoids, or developmental-pathway transcriptomic signatures may indicate that pregnancy and early-life developmental stages represent periods of increased susceptibility relative to healthy adults. Such information can be incorporated directly into subgroup-specific risk characterization and support targeted risk-management measures. Similar approaches are already employed in other public-health domains; for example, food allergen management relies on identifying susceptible populations and communicating risks specifically to those groups rather than applying blanket restrictions to the entire population. Extending this principle to chemical assessment would allow probabilistic NGRA to provide more proportionate and protective regulation for susceptible and vulnerable individuals.

### 3.3 Health impact pathways (HIPs)

**FIGURE 2:**
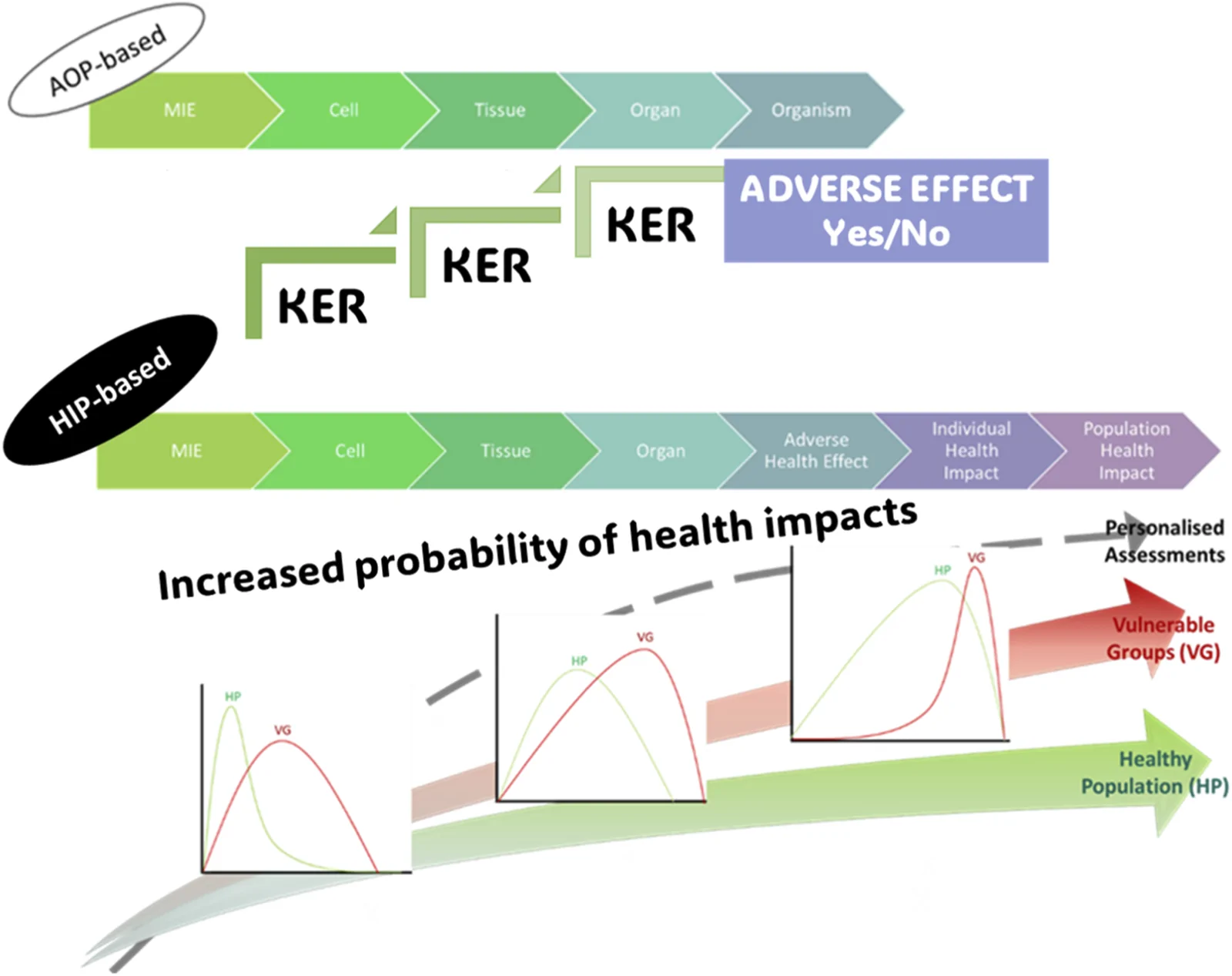
Linking Adverse Outcome Pathways (AOPs) to Health Impact Pathways (HIPs). This figure illustrates the conceptual advancement from mechanistic toxicology toward population-relevant risk prediction. The He*alth Impact Pathways (HIPs)* integrates molecular initiating events (MIEs), cellular and organ-level responses, and adverse health effects within a probabilistic architecture that connects *Adverse Outcome Pathways (AOPs)* to measurable health indicators. AOPs are built on a binary approach, with sequential Key Events connected by Key Event Relationships (KER) that determine if the effects progress or not to the next level and finally to an adverse outcome, accounting only for direct effects. In contrast, HIPs support a continuum approach, with KEs aggregated as physiological alterations that increase the probability of (in)direct impacts on the health status, accounting of structural and physiological compensations, that halt the AOP progress but reduce resilience increasing the likelihood for related effects. The figure highlights that HIPs differentiate responses between *healthy populations (HP)* and *vulnerable groups (VG)*, the latter including individuals with pre-existing health conditions or higher exposure burdens. As molecular and cellular perturbations propagate along biological levels of organization, the divergence between these populations becomes increasingly evident. HIPs translate toxicologic understanding into probabilistic public health indicators, such as increased risk of healthcare attention or specific mortality odds frequently used in epidemiological studies, establishing links between both scientific approaches. This predictive, human-relevant framework for probabilistic Next-Generation Risk Assessment (NGRA) represents the foundation for personalized assessments based on the genetic background and the environmental epigenetic adaptations of each individual.

As AOP networks mature providing more detailed maps of how molecular events lead to adverse outcomes, our understanding of the biological mechanisms of non-infectious diseases continues to expand (Vinken, 2013). This triggers a new concept for using adversity pathways in health risk assessments, bridging toxicology and epidemiology, the Health Impact Pathways (HIPs) (Figure 2). We define a Health Impact Pathway (HIP) as a quantitative framework that links MIEs, key biological perturbations, physiological adaptations, susceptibility and vulnerability factors, and exposure distributions to measurable population-level health indicators. Unlike traditional AOPs, where progression is primarily evaluated through movement toward a predefined adverse outcome, HIPs consider that perturbations occurring at intermediate biological levels may already contribute to detectable public health impacts through altered resilience, reduced compensatory capacity, or increased susceptibility to secondary stressors. Therefore, within the HIP approach, impacts at MIE and KE level increases the possibility for health impacts, and therefore can be associated with an increased probability of impacts at population level. We have explored this approach for atmospheric pollutants, combining KEs in an AOP network into mechanistic pathways linked to measurable health impacts, as those used in epidemiological studies (Pallarés Porcar et al., 2024). For example, KEs associated to structural changes can be compensated and not affecting the respiratory function under normal conditions, but nevertheless increase the possibility of respiratory failure in connection with respiratory diseases or other stressors. Thus, while these KEs are not associated to adversity in the AOP, they are expected to increase public health indicators such as the need for medical respiratory care or even respiratory mortality rates at population level. The probability for these impacts is higher for groups with respiratory vulnerability such as asthmatics. Thus, HIPs operationalize AOP networks into quantitative risk functions that regulators can use to predict the likelihood and severity of health impacts under real-world exposure scenarios.

As an illustrative example, exposure to NO_2_ may activate inflammatory pathways represented within an AOP network. Although many exposed individuals may not progress to a clinically adverse respiratory outcome, persistent activation of inflammatory key events may reduce physiological resilience and increase the probability of asthma exacerbations, emergency room visits, or respiratory hospitalizations. Within the HIP framework, these mechanistic perturbations can therefore be translated into probabilistic health impact functions linked directly to epidemiological indicators (Pallarés Porcar et al., 2024).

The construction of a HIP involves five sequential steps: (1) identification of relevant MIEs and KEs through AOP networks; (2) characterization of quantitative relationships between KEs and physiological alterations; (3) identification of factors modifying susceptibility and vulnerability; (4) integration of exposure distributions with mechanistic response distributions; and (5) translation of resulting probabilities into population-level health indicators suitable for regulatory decision-making.

#### 3.3.1 What differentiates HIPs from existing frameworks?

HIPs build upon, but are distinct from, existing toxicological and public health frameworks. AOPs describe causal biological chains linking molecular initiating events to adverse outcomes. qAOPs introduce quantitative relationships between key events, while AOP networks capture interactions among multiple pathways (Villeneuve et al., 2018). Aggregate Exposure Pathways (AEPs) describe how external exposures reach target sites. Health Impact Assessments evaluate the public health consequences of policies or environmental changes. HIPs integrate elements of these frameworks but focus specifically on translating mechanistic biological perturbations into quantitative estimates of population-level health impacts. The central innovation of HIPs is the use of mechanistic key event information to generate probabilistic health-impact functions that can estimate changes in epidemiological outcomes such as disease incidence, healthcare utilization, or mortality.

## 4 Implementing probabilistic NGRA

**FIGURE 3:**
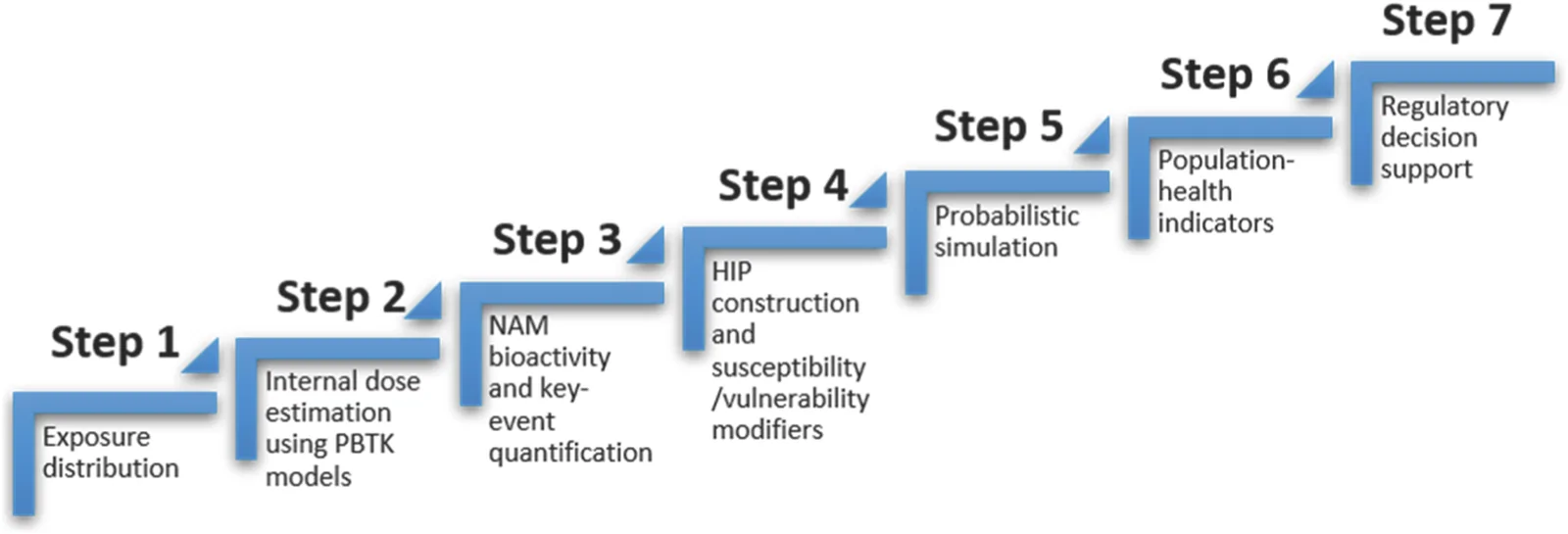
Proposed workflow for implementing probabilistic Next-Generation Risk Assessment (NGRA). The framework integrates exposure distributions, physiologically based toxicokinetic (PBTK) modelling, NAM-derived bioactivity and key-event quantification, Health Impact Pathway (HIP) construction, probabilistic simulation, and population-health indicators to generate regulatory decision-support outputs. The workflow illustrates how mechanistic and exposure information can be progressively translated into population-relevant risk estimates while accounting for variability, uncertainty, susceptibility, and vulnerability.

Transitioning from a deterministic to a probabilistic risk assessment framework requires not only conceptual change but also methodological innovation and regulatory adaptation. Implementation must ensure that probabilistic principles are embedded across all components of risk assessment, from data collection to regulatory decision-making. Figure 3 illustrates a proposed workflow for implementing probabilistic NGRA, integrating exposure assessment, mechanistic NAM evidence, HIPs, probabilistic modelling, and regulatory decision support within a single framework.

### 4.1 Data integration and FAIR principles

The success of probabilistic NGRA relies on integrating diverse data streams (mechanistic, exposure, biomonitoring, and epidemiological) into a coherent framework. To achieve this, adherence to FAIR (Findable, Accessible, Interoperable, Reusable) principles is essential (Wang and Savard, 2023). FAIR-compliant data infrastructures enhance transparency, harmonization, and cross-sector comparability, accelerating regulatory uptake of probabilistic methods (Bhat and Wani, 2025; Ugochukwu and Phillips, 2024).

Implementation of probabilistic NGRA will require several categories of mechanistic evidence, including: (i) identification of molecular initiating events; (ii) quantitative key-event relationships; (iii) concentration-response relationships from NAM systems; (iv) toxicokinetic and physiologically based kinetic model parameters; (v) biomarkers of exposure and effect; (vi) susceptibility- and vulnerability-related determinants; and (vii) epidemiological indicators used for HIP calibration and validation.

Key data categories requiring FAIR implementation include NAM-derived bioactivity data, toxicokinetic datasets, exposure models, human biomonitoring results, epidemiological outcome data, AOP and HIP annotations, uncertainty metadata, model parameter libraries, and computational code supporting probabilistic simulations. Ensuring interoperability across these data streams is particularly important because probabilistic NGRA depends upon integration of evidence originating from diverse scientific disciplines.4.2 Addressing Uncertainty & Variability.

Another key feature is the explicit treatment of uncertainty and variability while treating the data. Making uncertainty visible and quantifiable increases scientific robustness, supports public trust, and enables proportionate regulatory action (OECD, 2019). In TRA, intraspecies variability is assessed as a default value of 10 times or a combination of toxicokinetic and toxicodynamic subfactors (Dorne et al., 2005). Our approach not only uses the full distribution, but also uses NAM mechanistic data to identify which groups may be more vulnerable due to toxicokinetic or toxicodynamic differences vs. healthy individuals (Fernández-Martín and Tarazona, 2025). Health statistics suggest that about one-half of adults in developed countries have at least one chronic disease, and in some cases such as oncological patients, the vulnerability is linked to effects such as immunotoxicity poorly addressed by animal studies.

A practical challenge is that many high-throughput screening assays generate binary activity calls or AC_50_ potency values rather than continuous response functions. Several approaches may be used to address this limitation, including concentration-response modelling, benchmark concentration estimation, Bayesian uncertainty distributions, or integration of multiple assays into aggregate key-event scores. Such approaches can provide continuous probabilistic inputs suitable for implementation within HIP-based frameworks.

### 4.2 Tiered and scalable workflows

Probabilistic NGRA requires tiered, scalable workflows that can adapt to chemical context, data availability, and regulatory needs (Fernandez-Agudo and Tarazona, 2025; Leonard and Tan, 2019). Early tiers can rely on computational screening and *in silico* models, while higher tiers incorporate NAMs, AOP/HIP modeling, and probabilistic simulations. Pilot projects (e.g., Van Gerwen and Gorris, 2004) illustrate the feasibility of phased implementation, which facilitates gradual regulatory adoption and reduces reliance on animal testing.

An example implementation pathway is shown in Figure 3, illustrating how exposure distributions, mechanistic evidence from NAMs, HIP development, and probabilistic modelling can be integrated into a tiered workflow culminating in population-health indicators and regulatory decision support. The workflow illustrated in Figure 3 demonstrates how individual components may be progressively incorporated within a tiered implementation strategy, allowing adaptation to both data availability and regulatory needs.

### 4.3 Regulatory and stakeholder alignment

International misalignment remains a barrier (Reddy et al., 2023) to NGRA adoption. Initiatives such as ILMERAC, PARC, and the EC Roadmap for phasing out animal testing demonstrate steps toward harmonization, but stronger cross-jurisdictional cooperation is needed.

Stakeholder collaboration across regulators, industry, academia, and civil society is critical. Clear visualization tools such as risk distributions, exceedance probabilities, confidence intervals are essential for regulatory interpretation and communication (Sewell et al., 2024). Validation studies further ensure credibility and foster trust.

#### 4.3.1 Interpretation of probabilistic outputs in decision-making

Probabilistic NGRA does not necessarily replace regulatory decision criteria but provides additional information for decision-making. Outputs may include the probability of exceeding predefined health-effect thresholds, subgroup-specific risk distributions, expected changes in health indicators, and confidence intervals surrounding risk estimates. Regulatory decisions may be informed by both the magnitude of risk and its distribution across susceptible and vulnerable populations. Such outputs can support prioritization, targeted interventions, population-specific protections, and iterative reassessment as new evidence emerges. Emerging regulatory initiatives already reflect elements of this approach. For example, under implementation activities supporting the Water Framework Directive (WFD), ECHA and European partners (JRC, Joint Research Centre: Institute for Health and Consumer Protection Environment Agency et al., 2010) are increasingly considering exposure modelling, monitoring information, and population exposure dynamics to support the identification, prioritisation, and management of substances of concern. These developments suggest that regulatory decision-making is gradually evolving beyond reliance on single deterministic metrics toward more integrated and evidence-rich approaches.

### 4.4 Economic and ethical considerations

While initially resource-intensive, probabilistic NGRA offers long-term cost and time savings by reducing reliance on animal testing and prioritizing chemicals through predictive models (Luechtefeld et al., 2018). The framework also aligns with ethical imperatives, supporting the EC objective of phasing out animal testing while improving human relevance and public health impact.

## 5 Limitations and implementation challenges

Despite its potential advantages, probabilistic NGRA faces several scientific and regulatory challenges. First, quantitative mechanistic datasets remain unavailable for many toxicity pathways, limiting model development and calibration. Second, uncertainties remain when linking early molecular perturbations to population-level health indicators. Third, validation frameworks for probabilistic NAM-based models are still evolving and regulatory acceptance may vary among jurisdictions. Fourth, communicating probability distributions to decision-makers remains more complex than communicating threshold-based metrics. Finally, not all biological perturbations observed in NAM systems necessarily translate into adverse health outcomes, creating a risk of overinterpreting mechanistic signals. These limitations highlight the need for continued model development, transparent uncertainty characterization, and iterative validation strategies.

## 6 Toward a transformative NGRA paradigm

Revisiting Paracelsus’ principle that “only the dose makes the poison,” this work proposes a reinterpretation for the second half of the twenty-first century, shifting from the dose alone to the complex interaction between exposure, individual biology, and population variability. The aspiration for the coming decades is that risk assessment will evolve into a science capable of linking public health outcomes not only to levels of chemical exposure but also to the intrinsic characteristics, susceptibilities, and real-life conditions that define each population group.

The implementation of probabilistic NGRA marks an important development in chemical risk assessment. Unlike deterministic models, it integrates mechanistic data, variability, and uncertainty into a coherent framework capable of predicting not only whether harm may occur, but also how likely it is, for whom, and under what conditions.

HIPs are pivotal in this transformation. By linking molecular events to population-level health outcomes through probabilistic functions, HIPs enable regulators to interpret adversity as a continuum of risk rather than a binary threshold. HIPs connect KEs with the likelihood of measurable public health indicators. This, offers new options for validating NGRA outcomes, bridging toxicology with epidemiology, creating a truly relevant framework for public health.

The greatest advance of probabilistic NGRA is its ability to identify and characterize risks for susceptible and vulnerable populations. This capacity anticipates the next logical step, Personalized Risk Assessment (PRA), where risk estimates are tailored to individual exposomes and biological profiles.

Thus, probabilistic NGRA is not an endpoint but a necessary bridge.

- From TRA → NGRA → Probabilistic NGRA → PRA.
- From static thresholds → probabilistic risk distributions.
- From animal-based extrapolations → human-relevant, mechanistic predictions.
- From generic safety factors → identification of susceptible and vulnerable groups.

By embedding probabilistic reasoning with the use of NAMs and HIPs at its core, NGRA can deliver more accurate, ethical, and actionable risk assessments. This paradigm shift enhances public health protection, supports regulatory innovation, and lays the foundation for a future where risk assessment is personalized, mechanistic, and fully probabilistic.
